# Ethanol-Induced Effects on Sting Extension Response and Punishment Learning in the Western Honey Bee (*Apis mellifera*)

**DOI:** 10.1371/journal.pone.0100894

**Published:** 2014-07-02

**Authors:** Manuel A. Giannoni-Guzmán, Tugrul Giray, Jose Luis Agosto-Rivera, Blake K. Stevison, Brett Freeman, Paige Ricci, Erika A. Brown, Charles I. Abramson

**Affiliations:** 1 Laboratory of Behavioral Biology and Comparative Psychology, Oklahoma State University, Stillwater, Oklahoma, United States of America; 2 Department of Biology, University of Puerto Rico Rio Piedras, San Juan, Puerto Rico; Virginia Commonwealth University, United States of America

## Abstract

Acute ethanol administration is associated with sedation and analgesia as well as behavioral disinhibition and memory loss but the mechanisms underlying these effects remain to be elucidated. During the past decade, insects have emerged as important model systems to understand the neural and genetic bases of alcohol effects. However, novel assays to assess ethanol's effects on complex behaviors in social or isolated contexts are necessary. Here we used the honey bee as an especially relevant model system since bees are typically exposed to ethanol in nature when collecting standing nectar crop of flowers, and there is recent evidence for independent biological significance of this exposure for social behavior. Bee's inhibitory control of the sting extension response (SER) and a conditioned-place aversion assay were used to study ethanol effects on analgesia, behavioral disinhibition, and associative learning. Our findings indicate that although ethanol, in a dose-dependent manner, increases SER thresholds (analgesic effects), it disrupts the ability of honey bees to inhibit SER and to associate aversive stimuli with their environment. These results suggest that ethanol's effects on analgesia, behavioral disinhibition and associative learning are common across vertebrates and invertebrates. These results add to the use of honey bees as an ethanol model to understand ethanol's effects on complex, socially relevant behaviors.

## Introduction

Ethanol is thought to be an evolutionarily important cue for humans to find mature fruits [1]. However, today ethanol availability and effects on complex behavior result in negative individual and social outcomes [2]. Even a single episode of excessive ethanol drinking can lead to negative outcomes such as violent crimes, aggression and increased risk of accidents, injury and death [3], [4]. These negative outcomes are linked to ethanol's impact on complex behaviors such as ability to induce behavioral disinhibition [5], sedation [6], [7], analgesia [8] and learning and memory deficits [9]. The mechanisms underlying these effects require further investigation in humans and model organisms.

The rationale behind the current experiments is to investigate the analgesic properties of ethanol in the honey bee. Analgesia refers to the suppression of signaling of aversive stimuli to the brain such that nociception is reduced or blocked. In humans, there was first anecdotal evidence about the ability of ethanol to influence pain [10]. Later experimental studies supported that drinking ethanol affects pain perception [11]–[15].

Currently the analgesic effects of ethanol are well established [16], [17]. While “pain” is a psychological concept [18] aversive conditioning studies with bees show that they will work to turn off an aversive stimulus and to actively avoid a cue that has been previously paired with such a stimulus [19]–[21]. The use of honey bees can also help a researcher focus on behavioral response to aversive stimuli [22].

In insect models, acute exposure to ethanol, and its behavioral and molecular effects are studied [23]. Honey bees differ from other insect models by providing an experimentally accessible social organism to study acute ethanol exposure effects [24]. Our work has revealed that ethanol consumption has effects on locomotor activity, social interactions, stress signals, performance, and even reproduction [25]–[28]. The bee model is especially relevant since bees are naturally exposed to ethanol due to fermentation in standing nectar crops of flowers they visit [29], [30]. We do not know if bees seek ethanol, however foragers produce the pheromone ethyl oleate from ingested ethanol, which regulates social behavioral development [31]–[33]. In the bee, the molecular substrates of potential ethanol pathophysiological action are also present [34], [35], and indeed ethanol impairs appetitive conditioning [36], [37]. However, studies on aversive conditioning in bees are recent [38] and earlier studies focused on appetitive stimuli [19]–[21], [39].

Sting response is the delayed, last stage in colony defense, and it is costly both for the individual and the colony [40]–[42]. Sting response against vertebrates often result in the stinger detaching from the abdomen, and eventual death of the individual worker [40]. Alarm pheromone is released in the process and more of the colony work force engages in the potential self-sacrificial act of aggressive colony defense. Ecological conditions shape the response, with tropically adapted bees responding sooner and at greater numbers than the temperate bees [41], probably due to a trade-off between work force allocated to food collection vs. aggressive defense [41]–[44]. There are also individual differences such as higher expression of genes related to signaling, and lower expression of genes related to metabolic processes in bees engaged in defense in comparison to bees engaged in other jobs [45]. Individual worker bees demonstrate a threshold response to aversive stimulus, indicative of inhibition of stinging behavior [46], [47]. In addition, the sting response circuit of the honey bee responds to opioids [48], [49], and to biogenic amine modulation [38].

The study of ethanol effects on the bee sting extension response and aversive conditioning to electric shock could lead to further mechanistic understanding of alcohol induced analgesia, behavioral disinhibition, and learning and memory deficits: analgesia could be adaptive. For instance, in the event of a lesion during a predator-prey interaction, analgesia may allow the organism to focus on escaping and surviving despite a serious injury [50]. Ethanol is known to induce analgesia in a wide range of mammals [17], [51], [52]. The precise mechanisms underlying the analgesic effects of ethanol are not fully understood but it is clear that both opioid and non-opioid mechanisms are involved [16], [53]. Behavioral disinhibition refers to the release of natural impulses (e.g. sexual, appetitive, social or aggressive drives) from the control of innate or learned inhibitions [54]. Ethanol and other addictive drugs have the common effect of inducing behavioral disinhibition [55], [56]. Behavioral disinhibition is thought to underlie the social and anti-social effects of ethanol such as increased social interactions (i.e. social lubricant), risk-taking and aggressive behavior [54], [57].

Although the mechanisms by which ethanol induces behavioral disinhibition remain unclear, accumulating evidence indicates that alterations in serotonergic and dopaminergic neurotransmitter systems within specific brain regions are central to this phenomenon [6], [56], [58], [59]. Learning and memory effects of ethanol consumption are highlighted by ethanol-induced “blackouts” [60]. High doses of ethanol disrupt short and long-term memory as well as explicit, intentional and effortful memories [61]–[64]. The mechanisms underlying ethanol effects on learning and memory involve multiple brain areas and the interaction with molecules regulating synaptic plasticity (e.g. NMDA receptor and GABA [65]–[68]).

We used a sting extension response (SER) assay [19], [39] and a recently developed aversive conditioning assay (electric shock avoidance—ESA) to examine: 1) *analgesia*, as indicated by increased shock threshold before sting extension across increasing doses of oral ethanol administration, and 2) *learning and memory deficits* using the aversive, non-appetitive place conditioning assay[20], [21]. We assayed ESA (12V, 50mA DC electrical shock) when the subject is on the designated color for shock under various alcohol treatments.

## Results

### Experiment 1: Ethanol effects on sting extension response (SER)

**Figure 1:**
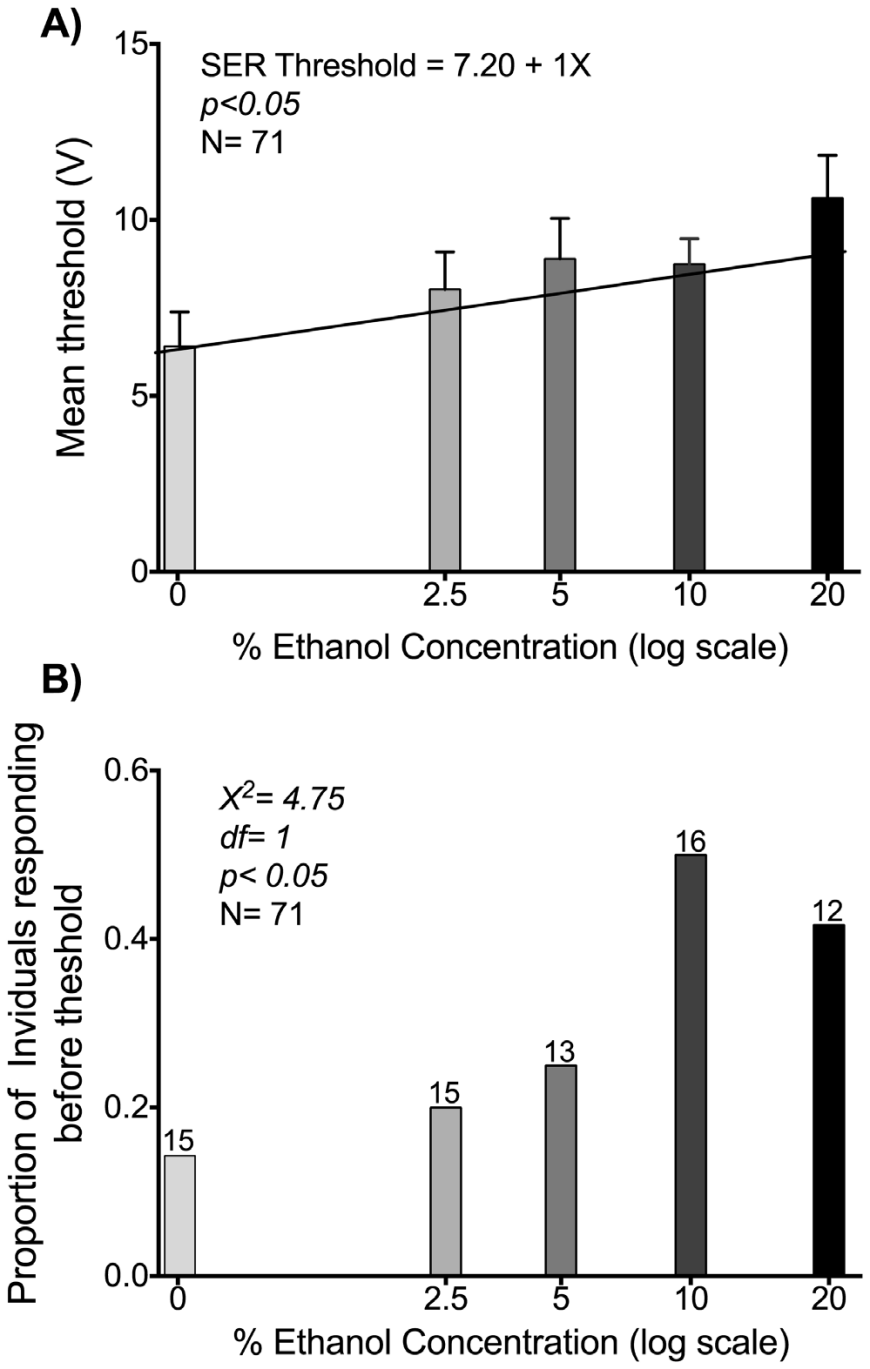
Ethanol increases sting extension response (SER) threshold of honey bee foragers A) Mean voltage threshold for SER for bees in 0%, 2.5%, 5%, 10%, and 20% ethanol treatment groups. Increased ethanol concentration treatment is associated with increased voltage threshold for SER. Linear regression of mean threshold response is statistically significant (*p<0.05*). B) Sting response before threshold. Proportion of individuals responding before threshold to the stimulus increased as ethanol concentration increased. Comparison between concentration groups using *Pearson*'*s X^2^* revealed significant differences across the groups (*X^2^ = 4.75, df = 4, p<0.05*). Numbers above bars are bees tested at each ethanol concentration.

To determine if ethanol alters the response to a noxious electrical stimulus in honey bees we used the SER assay. We hypothesized that if the analgesic effects of ethanol are conserved in honey bees then the voltage threshold required to elicit a stinging response would be higher in ethanol fed bees. Mean sting extension response (SER) threshold for the bees in 0%, 2.5%, 5%, 10%, and 20% ethanol concentration treatment groups is plotted (ethanol concentrations in log scale) in Figure 1A. We previously measured hemolymph ethanol levels using GC-MS after feeding honey bees different concentrations of ethanol in 10 µl 1.5 M sugar solution, as in the current study. For instance, 60 mM hemolymph ethanol concentration is achieved in 10 minutes post-consumption of the complete 10 µl volume of 5% ethanol concentration in solution, presented using a micropipette to a harnessed bee. The levels are maintained within statistical limits for up to 8 hours [37]. Each bee was captured, anesthetized (on ice), harnessed, and after recovery and feeding of the sugar solution (∼20 min), and the 10 min waiting period, was tested for sting response threshold in linear steps of 1 V up to 30 V maximum. Sting response threshold is the first of 3 consecutive voltage levels at which the harnessed bee responds by extending the stinger. Increased ethanol levels corresponded to increased response threshold. The bees in the no alcohol (0%) group had ca. 7 V average SER threshold voltage whereas the bees at the highest ethanol concentration treatment (20%) had a 10.75 V average SER threshold. The regression of SER threshold voltage on ethanol concentration was statistically significant (see Figure 1A).

Generally, the sting response threshold was also the first voltage level when sting extension response was observed. However, especially in the higher ethanol concentration groups, individuals extended their stinger at one voltage level, but did not extend their stinger in the following voltage level. Starting at a higher voltage level these bees also responded in consecutive voltage levels. The first, or first few “erratic” responses are identified as “sting response before threshold”. We decided to examine the relation of this erratic SER before threshold. Proportion of bees responding with SER before the threshold voltage in each group is plotted in Figure 1B. Increased ethanol concentration resulted in larger proportion of individuals giving SER before the response threshold, reaching half of all individuals at 10% alcohol concentration. A statistically significant trend was detected for increased proportions of individuals demonstrating SER before the threshold voltage with increasing treatment ethanol concentration (X^2^ = 4.75 *df* = 1 *p*<0.05, Figure 1B).

### Experiment 2: Ethanol effects on ESA conditioning

To determine the effects of ethanol on aversive learning in honey bees we used ESA conditioning. Due to the observed analgesic effects, we first determined if 12 volts could sustain aversive conditioning in our assay (experiment 2.1) and then tested the effects of ethanol (experiment 2.2).

#### 1) Optimal conditioning voltage: Effectiveness of 12 volts as shock punishment in aversive conditioning

The results of the SER threshold experiments suggested to us that to prevent reduced nociception that could interfere with our later experiments on the influence of ethanol concentration on learning we should use a shock level above the threshold (12 V) for the ESA conditioning experiments. We first tested if this high voltage would sustain aversive conditioning, 18 experimental bees and 18 yoked control bees were trained in pairs. Training sessions lasted ten minutes and involved a shock of 12 volts on the shock side (yellow or blue, in counterbalance experiments) of the shuttle box. No ethanol was used in the experiment. An analysis of the total number of responses demonstrated that bees in both groups had similar overall locomotor activity (Figure S1).

We examined the mean amount of time spent on the shock side of the shuttle box by the experimental and yoked bees. An analysis of variance did not yield a significant color effect, *F* (1,9) = 2.21, *p* = 0.17, but did yield a significant time effect, *F* (9,81) = 3.75, *p*<0.01 (Figure S2). Since the counter balanced design did not detect color differences, our subsequent analyses did not include color as a variable.

**Figure 2:**
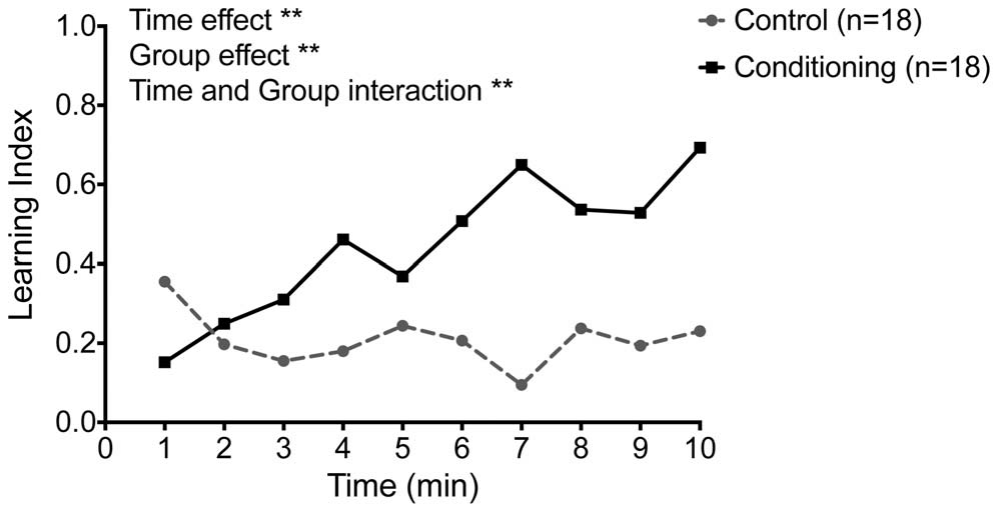
ESA conditioning in honey bee foragers using 12 volt, 50 Conditioned individuals (solid line) showed a greater increase in learning index over time compared to the control group (hedged line). Two-way ANOVA resulted in a significant time effect (*F* (9,153) = 4.05, *p<0.01*), significant group effect (*F* (1,17) = 21.02, *p<0.01*), and significant time and group interaction effect (*F* (9,153) = 5.74, *p<0.01*).

Figure 2 shows the learning index changes for conditioning and control group when 12 V shock punishment was used for conditioning. Two-way repeated measures analysis of variance (Conditioning: Yoked vs. Experimental; and Time: minute 1–10) results yielded significant group effect, *F*(1,17) = 21.02, *p*<0.01, time effect, *F*(9,153) = 4.05, *p*<0.01, and Group and Time interaction, *F*(9,153) = 5.74, *p*<0.01. The punishment level of 12 volts successfully supported conditioning as indicated by significantly different change in learning index for the conditioning group and the control group (see Figure 2). The increasing learning index for experimental bees shows decline in the amount of time spent on the shock side by the experimental bees relative to the yoked control bees.

#### 2) Ethanol Conditioning

**Figure 3:**
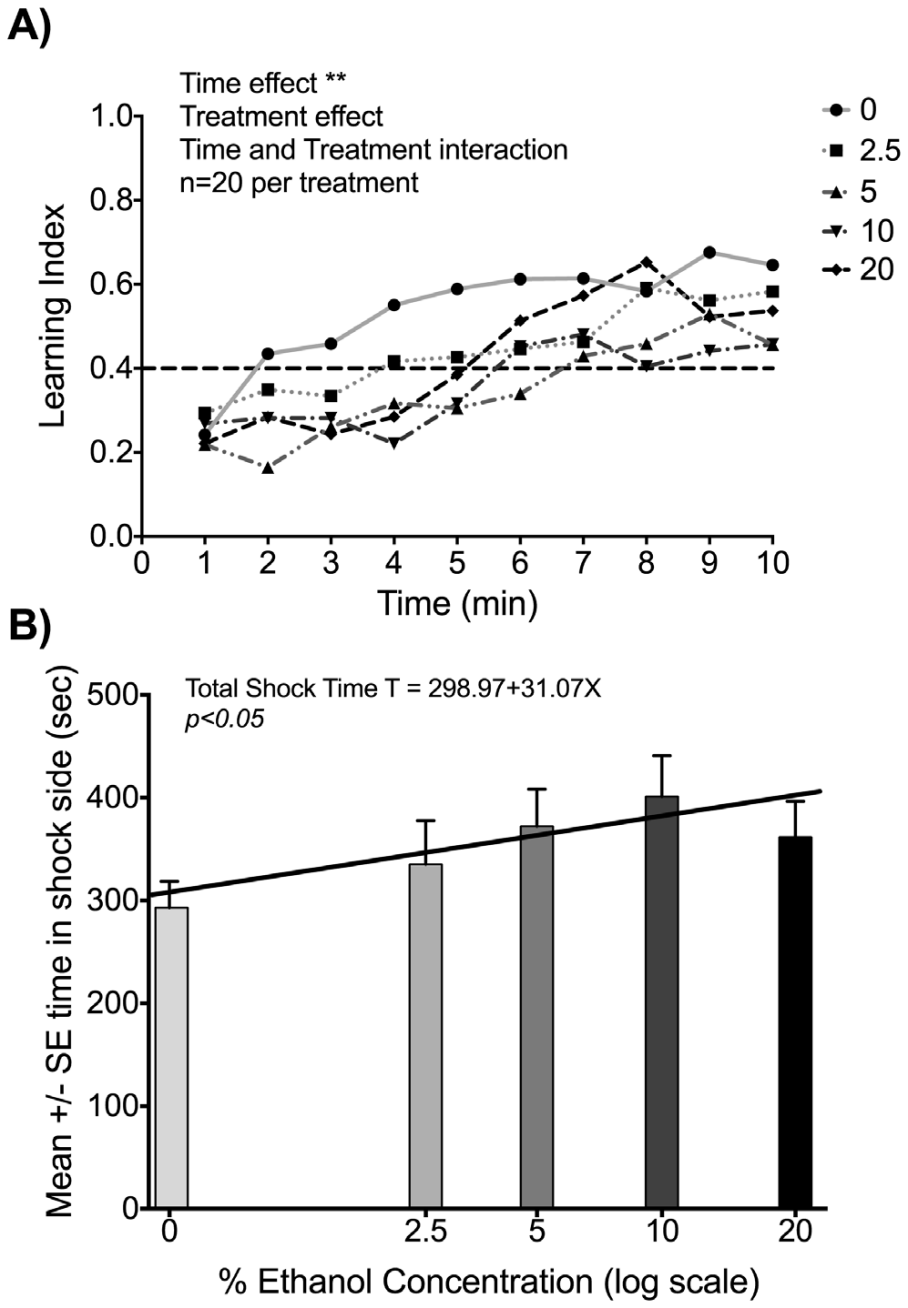
Ethanol treatment affects ESA conditioning in honey bee foragers. **A)** Learning index over time for 0%, 2.5%, 5%, 10%, and 20% ethanol treatment groups (see legend on plot). ANOVA analysis resulted in non-significant treatment effect (*F* (36,855) = 0.59, *p = 0.97*), significant time effect (*F* (9,855) = 4.60, *p<0.001*) and non-significant time and treatment interaction (*F* (4,95) = 1.51, *p* = 0.*21*). These results demonstrate that even with ethanol administration bees were able to learn to avoid color associated with 12 V punishment. However, early phase of training differs across ethanol treatment groups, at 5% or higher ethanol treatment longer training time is required for similar improvement in learning index (see Results). **B)** Mean and SE of time spent in shock side for 0%, 2.5%, 5%, 10%, and 20% ethanol treatment groups. Increased ethanol concentration treatment is associated with an increase in time spent in the shock side during the ESA conditioning, indicating lower learning performance. Linear regression of mean time spent in the shock side is statistically significant (*p<0.05*).

A continuous ten minute training session was used to train 100 bees. Twenty bees were randomly assigned to each of the 0%, 2.5%, 5%, 10%, and 20% ethanol concentration groups. In light of absence of a color effect on conditioning and movement rate in Experiment 2, a 12 volt shock was used on the blue side of the shuttle box only. The mean number of responses for the bees in the 0%, 2.5%, 5%, 10%, or 20% ethanol concentration treatment groups was not different (Figure S3). A 5 (Group: 0%, 2.5%, 5%, 10%, or 20% ethanol) by 10 (Time blocks: 10 one minute intervals) split-plot analysis of variance was conducted, with repeated measures on the Time factor for learning index. At the end of the ten minute training session, the bees were spending less time on the shock side compared to the beginning of the training session as indicated by greater learning indices achieved. Analysis of variance results showed no significant Treatment and Time interaction, *F* (36,855) = 0.59, *p* = 0.97. The Group effect was also not significant, *F* (4,95) = 1.51, *p* = 0.21. There was, however, a significant Time effect, *F* (9,855) = 4.60, *p*<0.01 (Figure 3A). These results show that even with ethanol administration bees were able to learn to avoid color associated with punishment.

However, the differences in learning may be obscured with the general trend in improved learning performance for all groups, for instance due to overtraining (increase in learning index saturates after about the first 7 minutes of training for all groups). One important measure in place preference learning is the total time spent in the alternate place [20], [69]. For total amount of time spent in the shock area, the treatment groups showed significant differences, with time increasing with increased ethanol concentration (Figure 3B). To test for differences in amount of learning achieved with training, we examined the training required in order to reach a 0.4 learning index. Indeed there are significant differences across treatment groups (N = 100; *df* = 4; F = 2.7408; *p*<0.05), and significantly greater training in minutes is required for treatment groups at 5% and greater ethanol concentration as indicated by post hoc comparisons (Treatment group: mean training in minutes to 0.4 learning index ± SE: 0%: 3.22±0.83; 2.5%: 3.25±0.83; 5%: 5.38±0.83; 10%: 5.58±0.74; 20%: 5.97±0.76).

## Discussion

The overall significance of these results is the demonstration that ethanol influences socially relevant complex behavior in honey bees, in ways comparable to ethanol effects in other organisms. Ethanol treated bees were less sensitive to shock as indicated by increased SER threshold, yet they were more erratic in response, as indicated by isolated SERs given before the measured threshold. In addition, ethanol reduced aversive learning performance, similar to effects in other organisms, and similar to the effects on appetitive learning in honey bees [36]. The novel findings of analgesic and modulatory effects of ethanol on the aggressive response, and on aversive learning highlight the importance of honey bees as a socially relevant model for ethanol effects on behavior.

Ethanol-induced increase in SER threshold is consistent with the well-known analgesic effects of ethanol [16], [17]. Although we do not know the mechanism underlying this effect in honey bees, studies in mammalian systems have shown that ethanol-induced analgesia involves the activation of opioid receptors [53], [70]. Given that the analgesic effects of opioids are observed in honey bees and have been shown to increase SER threshold [49], it is tempting to speculate that ethanol-induced increase in SER threshold may be related to the opioid system. Alternately, ethanol effects may be due to changes in aminergic signaling and that in turn may influence the SER response and aversive learning (see [20], and reviewed in [38]). Although further studies are necessary to understand the underlying mechanisms, our findings suggest that the analgesic effects of ethanol are conserved in honey bees. Since ethanol is part of the natural honey bee diet, understanding the functional and ecological significance of ethanol's analgesic effects warrants further research.

Even though ethanol-treated bees require higher voltages to show consistent SER, they exhibited more erratic pre-threshold SER than non-treated bees. The probability to elicit a SER before the threshold voltage increases asymptotically to 50% at, or above, 10% ethanol concentration treatment (Figure 1B). This phenomenon resembles ethanol effects on human aggressive behavior i.e., intoxicated individuals tend to lose control and get into fights easier than when they are sober despite feeling numb from the alcohol [71]. In particular, in the case of honey bees, the effect of ethanol consumption on sting response prior to threshold stimulation could be interpreted as a lack of suppression of aggression. It is also known that free flying Africanized honey bees become more aggressive after exposure to ethanol [72]. This loss of control or lack of inhibition is a hallmark of ethanol intoxication in humans and is often referred to as behavioral disinhibition [6], [54], [57], [73]. Neuromodulatory mechanisms underlie behavioral disinhibition in vertebrates [6], [56] and the same neuromodulatory mechanisms are also involved in sting response behavior of honey bee workers [20], [38], [74]. Relation of serotonergic and dopaminergic pathways to ethanol needs to be examined in the honey bee.

We chose the 12 V punishment level to overcome interference between analgesic effect of ethanol and its possible effect on learning the punishment task. The ethanol effects on aversive conditioning in this study utilized an ESA, electric shock avoidance assay developed by several of us [20]. However, there were certain differences worth discussing in the protocol used here. Namely, we altered the punishment level to 12 V, instead of 6 V, and restricted training to one session of 10 minutes instead of two 5 minute sessions, divided by a 10 minute interval. Using this protocol we were able to demonstrate effective aversive conditioning at the 12 V shock level (see Figure S4 for 6 V 50 mA, 10 minute single training session).

The reason higher shock level was avoided by Agarwal and colleagues [20] in their study was to avoid interference in conditioning of bees by release of alarm pheromone by subjects in a multiple test where 10 individuals were tested simultaneously. Here we assayed only one individual in ethanol effect assays, and two individuals in the yoked control design. The interference across bees was circumvented because bees were in completely separate shuttle boxes [21]. The results also show that only training group bees showed a conditioned response and increased learning index over time.

The average high learning index, in this study was 0.7 and lower than the value near 1.0 previously reported [20]. The combination of massed and spaced training in Agarwal and colleagues [20] study may have resulted in the superior conditioning results reported. In this experiment, the use of yoked controls, and training one bee at a time, forced us to use a single 10 minute training period, instead of 20 minutes total (two 5 minute training periods divided by a 10 minute interval) for 10 bees trained in parallel [20]. The time required to train bees individually is 10 times greater in our protocol, and providing only one continuous training allows completion of the overall experiment within days, even with large number of individuals trained (e.g. 100 bees in ethanol treatment groups).

When we examined the effect of ethanol on conditioning in experiment 2 (0%, 2.5%, 5%, 10%, and 20% ethanol concentration groups) and we found all bees decreased their frequency of responding by the end of the training session compared to the beginning of the session, while the proportion of time spent on the safe side of the apparatus or learning index increased as trials continued. The general pattern of conditioning, and the learning index within the first minute was similar for experiment 2 and the yoked-control bees of experiment 1, indicating anesthesia prior to ethanol administration did not influence the ESA conditioning. Although the general pattern of conditioning was similar, alcohol did affect total time spent on the punishment side of the apparatus and also the amount of training required to reach similar learning indices. Increasing levels of ethanol treatment resulted in greater punishment time during conditioning.

The changes in the learning index are more erratic for bees treated with the highest ethanol concentration (20%). These effects did not alter the overall patterns, and inferences, yet demonstrate that at high concentrations of alcohol, bees became less able to function well in the apparatus. However, natural relevance of high levels of ethanol such as the 20% used here, are not clear since only ethanol concentrations of ∼5% or less are encountered by bees in nature.

Previous studies have found ethanol in standing nectar crops of flowers that bees visit in the field [75]. Therefore effects of ethanol demonstrated here, may influence social behavior of bees in nature in different contexts such as colony defense and assessing foraging rewards and risks of different resources. Examining effects of ethanol on the typical social behaviors of free flying honey bees may be an important future direction with insight into social biology of alcohol, and into the fit between rational models of social behavior, such as foraging choice [25], [76], [77].

One important area would be to examine individual differences in responses of bees to ethanol. Honey bees may help us understand interindividual differences seen in the spectrum from typical to pathological effects of ethanol consumption. Potential differences in molecular correlates of effects examined in this study could underlie differences in ethanol effects across individuals. Both across honey bee colonies and within the colony, individuals differ genetically (patrilineal origin) and also differ in experience, age and physiology (reviewed in [20], see also [40]–[48], [78], [79]). Analgesic effects suggest opioid pathway and neuromodulators as future subjects of investigation for ethanol effects. The reduced inhibition for SER may also indicate involvement of neuromodulators in ethanol effects (see [38], [74]). The changes in learning performance may indicate integrative processes such as ion channels important for neural communication. The influence of alcohol on the honey bee brain may provide clues to molecular correlates of social behavior and social effects of alcohol.

## Methods

### Bees

Bees were from *Apis mellifera* colonies maintained according to standard beekeeping methods at the apiary of Uludağ University, Bursa Turkey. These bees were previously identified as *Apis mellifera anatoliaca* subspecies based on morphological characteristics (see also [21]). Bees used in each study were all collected from a single, separate source colony to reduce potential effects of colony conditions on bees from different experimental groups.

Forager bees were collected individually at a feeder placed ∼100 m from the bee yard. Briefly, bees from two colonies were trained to forage at a feeder that provided a 50% sucrose solution, and was advertised to bees using lavender fragrance placed on a filter paper below the feeder (see [77]). For all experiments forager bees were collected at the feeder in individual vials. Bees were brought to the lab, anesthetized on ice in the vials, and used in further experiments as described later.

### Electric shock assay

To assay the behavior of the honey bee we used an apparatus that combines elements of a “Kolmes electric grid”, and a sting extension response assay [39], [80]. This apparatus is fully described in Agarwal et al. [20] and is essentially a shuttle box or choice chamber (see Figure S5). An individual bee is placed in a lane of the apparatus, isolated from others, and free to walk back and forth on an electrifiable grid (a manual switch controls the current). The bee cannot fly out because a Plexiglas panel is placed over the choice chamber providing only enough space for the bee to walk or stay on the grid surface. When the bee crosses to the half of the choice chamber with the underlying color cue that is associated with punishment, the observer closes the circuit and shock is applied to the bee. In yoked control experiments, two bees are placed in the apparatus in separate chambers, and shock is applied to both bees when the master bee enters the half with the color associated with shock. The time on shock or safe side is recorded for each bee by an observer other than the one applying the shock.

### Experiment 1: Ethanol effects on sting extension response (SER)

We examined the change in SER to electric shock across different intensities of shock and doses of oral ethanol administration. Five different alcohol sucrose (1 M) solutions were prepared, 0%, 2.5%, 5%, 10% and 20%. The subjects were cold anesthetized to quiescence (in collection tubes on ice-water for 2 minutes) and were then taped in the apparatus abdomen pointing upwards so the stinger can be seen. Immediately after bees were moving their appendages (e.g. responding by proboscis extension to sucrose stimulation on mouth parts, see [36]), they were subjected to the testing procedure, including the feeding (∼20 min), wait (10 min, required to reach stable ethanol titers in hemolymph), and SER measurement (1 V increments until 30 V, see below).

Each group started with twenty bees, which were fed 10 µl of solution ten minutes prior to the shock administration. Only bees that consumed all of the 10 µl solution were included in the test procedure. The shocks were administered as 1-second pulses for 3 seconds. After each 3 pulses, a 10 second interval was initiated to observe subject sting extension and also for the experimenters to change the shock voltage. The trial for each bee started at 1 volt and lasted until the bee displayed SER on 3 consecutive shock voltages or until we reached 30 volts (which was the power supply's limit). The voltage at the first of the three consecutive shock responses was recorded as the SER threshold. In rare cases, if animals do not to give the SER in 3 consecutive shock voltages, the second response voltage has been recorded as the threshold. Individuals that did not exhibit a clear SER threshold were excluded from further analyses. We also recorded the number of individuals from different treatment groups that responded with the SER before the SER threshold when a clear consecutive threshold was measured.

### Experiment 2: Ethanol effects on ESA conditioning

#### 1) Optimal voltage for ESA conditioning

As per results from Experiment 1, 12 volt shock punishment was chosen empirically, to ameliorate any reduced shock response due to analgesic effects of ethanol. 12 Volt shock is just above SER threshold of all treatment groups (see Results and Figure 1A). We tested whether this shock level based on the results of the shock-induced SER threshold experiment (12 V, 50 mA see above and results) would sustain conditioning in a continuous10-minute conditioning period.

For each shock level, a group of twenty bees were captured for shock conditioning. In the 12 V level, an additional group of twenty bees were captured to counter balance training colors. For bees in one group blue color was designated as the shock area (both for 6 V and 12 V) and for the other group the yellow color (only for 12 V level). Of the 20 bees in each group, ten were only to be shocked when they crossed into the color designated as the shock area (conditioning group). The other ten subjects were yoked controls and were shocked when the conditioning bee was being shocked regardless of the yoked bee's position in the apparatus. We used the learning index described in Agarwal et al. [20] (see also [21]), where time on the safe side that is greater than chance (>30 s in 1minute interval) is stated as a proportion (0 to 1) to plot a learning curve for both conditioning and control groups. For example, a bee that stays 45 seconds on the safe side during 1 min interval will be calculated to have a learning index of 0.5, this is calculated in the following manner: learning index =  (time on safe side – 1/2 time of interval)/(1/2 time of interval).

The experiment was run using only two bees at a time; one conditioning group bee and one yoked control bee [21]. The subjects were cold anesthetized to quiescence (in collection tubes on ice-water for 2 minutes) and placed in the apparatus in different shuttle boxes, and left for recovery (∼20 minutes). Every trial lasted 10 minutes, and we recorded the observations in these trials in one-minute bins or intervals. During these intervals one experimenter observed one of the bees in the apparatus and reported the number of responses and the time spent in each color per minute to a second experimenter. A third experimenter was in charge of supplying the shock each time the conditioning group bee crossed to the shock color. This experimenter was the only researcher who was not blind to which subject was from the conditioning group and which bee was the yoked control.

#### 2) Ethanol and conditioning

To investigate the effects of ethanol on punishment conditioning we used the ESA assay (12 V, 50 mA electrical shock). The avoidance was reinforced by shock when the subject is on the designated color for the punishment stimulus under various alcohol treatments. The subjects were cold anesthetized to quiescence and placed in the apparatus as described above. Five different 1 M sucrose solutions with differing alcohol concentrations were prepared, 0%, 2.5%, 5%, 10% and 20%. Each treatment group had twenty bees, which were fed 10 µl of solution ten minutes prior to the test. For this experiment blue was the only color designated as the shock area. The conditioning was performed as described above, except without the use of a yoked control.

## Supporting Information

Figure S1 **Locomotor activity (mean number of responses) of conditioned and yoked honey bee foragers were not different during ESA conditioning.** Eighteen out of 20 bees were trained in pairs using a yoked control design (2 bees were excluded from the study because they did not switch sides throughout the 10 minute assay) (12 volts, 50 mA). Training sessions lasted ten minutes and involved a shock of 6 volts on the blue side of the shuttle box. Two-way repeated measures analysis of the mean number of responses of yoked control (hedged line) and conditioned individuals (solid line) resulted in no significant differences between yoked and experimental bees, *F* (1,33) = 0.39, *p* = 0.84.
(TIF)

Figure S2 **The shock color did not influence ESA conditioning in honey bee foragers.** Individuals conditioned to avoid yellow colored area (solid line) showed a similar learning curve to bees conditioned to avoid shock in blue (hedged line) (12 volt, 50 mA. Two-way repeated measures analysis of variance (Conditioning: Yoked vs. Experimental; and Time: minute 1-10) results yielded non-significant color effect, *F* (1,9) = 2.21, *p* = 0.17 and a significant time effect, *F*(9,81) = 3.75, *p*<0.01.
(TIF)

Figure S3 **Locomotor activity of ethanol treated honey bee foragers is not different during ESA conditioning.** Mean voltage threshold for SER for bees in 0%, 2.5%, 5%, 10%, and 20% ethanol treatment groups. Increased ethanol concentration treatment is not associated with locomotor activity. Linear regression of mean number of responses is not statistically significant (*p = 0.17*).
(TIF)

Figure S4 **ESA conditioning using 6 volts, 50 mA punishment.** The shock conditions (6 V, 50 mA) described in the original study [20], resulted in ESA conditioning under the modified protocol used in this study (yoked control design and a single 10 minute training interval). Eighteen of 20 bees were trained in pairs using a yoked control design (2 bees were excluded from the study because they did not switch sides throughout the 10 minute assay). Training sessions lasted ten minutes and involved a shock of 6 volts on the blue side of the shuttle box. No ethanol was used for this experiment. Conditioned individuals (solid line) showed an increase in learning index over time compared to the control group (hedged line). Two-way repeated measures analysis of variance (Conditioning: Yoked vs. Experimental; and Time: minute 1–10) results yielded non-significant group effect, *F* (1,8) = 1.81, *p* = .22 and a significant time effect, *F* (9,72) = 2.54, *p*<0.01. In addition, the Group and Time interaction was significant, *F* (9,72) = 2.51, *p* = .02.
(TIFF)

Figure S5 **Diagram of the ESA apparatus.** This apparatus was previously described in Agarwal et al. (2011) [20]. For the present experiment only the first and last rows were used. Yoked bees (lane 1 or 10 in alternate runs) were shocked regardless of location each time the experimental bees (lane 10 or 1 in alternate runs) crossed into shock color.
(TIF)
