# Supplementary figures and images for: Ethanol-Induced Effects on Sting Extension Response and Punishment Learning in the Western Honey Bee (Apis mellifera)
Source: PLoS One. 2014 Jul 2;9(7):e100894. doi: 10.1371/journal.pone.0100894 (PMC4079248; doi:10.1371/journal.pone.0100894)

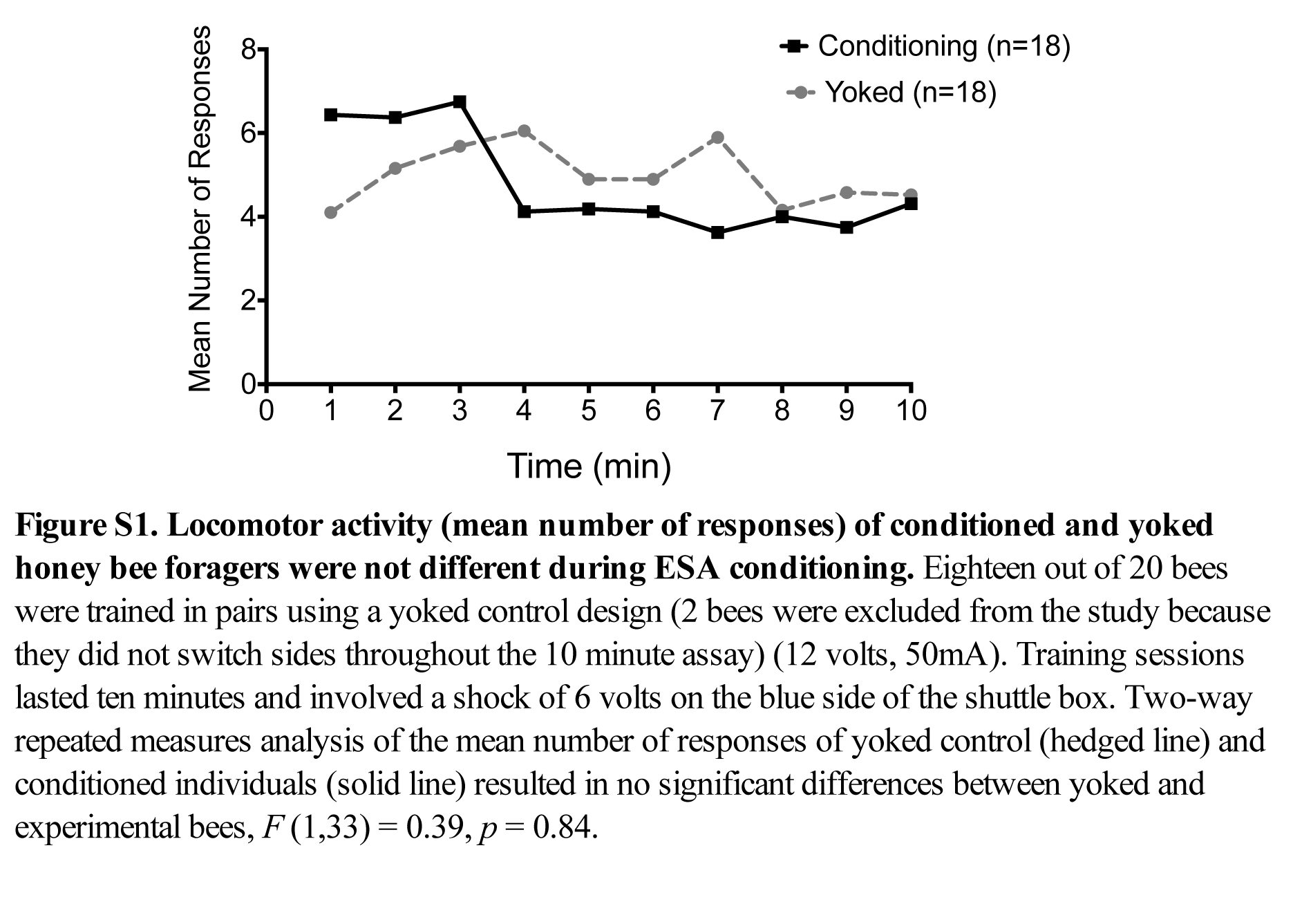

Supplement: Figure S1 — Locomotor activity (mean number of responses) of conditioned and yoked honey bee foragers were not different during ESA conditioning. Eighteen out of 20 bees were trained in pairs using a yoked control design (2 bees were excluded from the study because they did not switch sides throughout the 10 minute assay) (12 volts, 50 mA). Training sessions lasted ten minutes and involved a shock of 6 volts on the blue side of the shuttle box. Two-way repeated measures analysis of the mean number of responses of yoked control (hedged line) and conditioned individuals (solid line) resulted in no significant differences between yoked and experimental bees, F (1,33) = 0.39, p = 0.84. (TIF) [file pone.0100894.s001.tif]

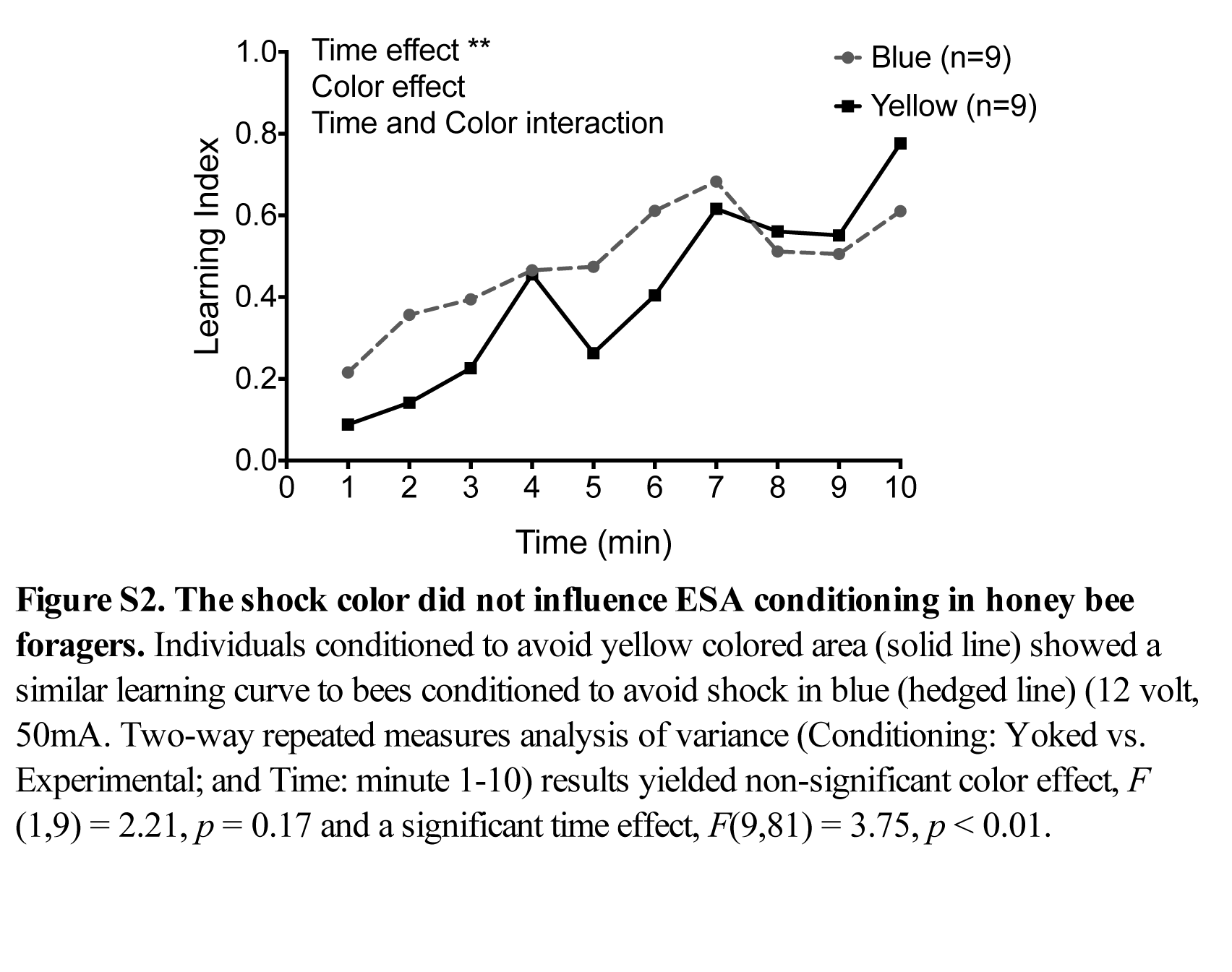

Supplement: Figure S2 — The shock color did not influence ESA conditioning in honey bee foragers. Individuals conditioned to avoid yellow colored area (solid line) showed a similar learning curve to bees conditioned to avoid shock in blue (hedged line) (12 volt, 50 mA. Two-way repeated measures analysis of variance (Conditioning: Yoked vs. Experimental; and Time: minute 1-10) results yielded non-significant color effect, F (1,9) = 2.21, p = 0.17 and a significant time effect, F(9,81) = 3.75, p<0.01. (TIF) [file pone.0100894.s002.tif]

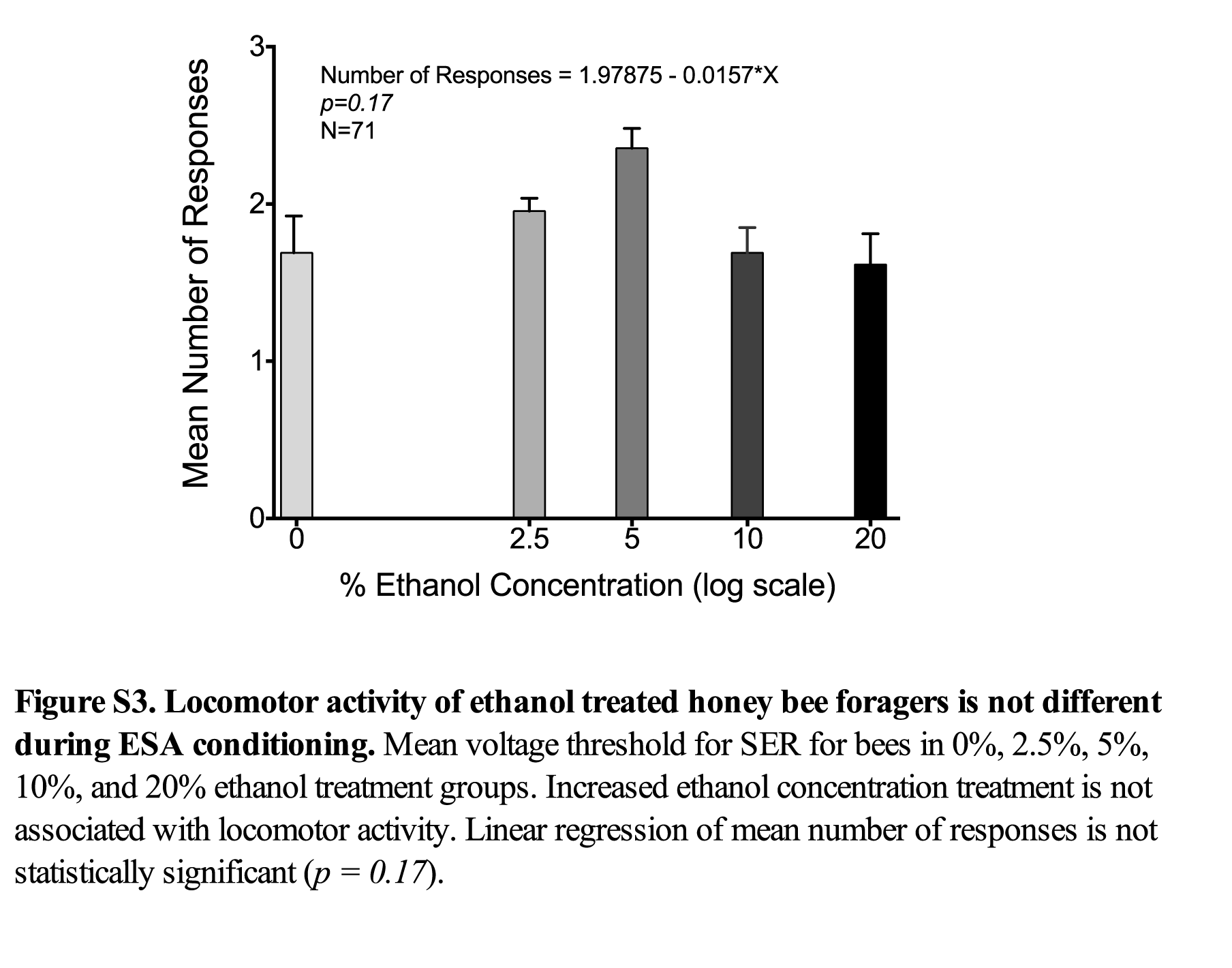

Supplement: Figure S3 — Locomotor activity of ethanol treated honey bee foragers is not different during ESA conditioning. Mean voltage threshold for SER for bees in 0%, 2.5%, 5%, 10%, and 20% ethanol treatment groups. Increased ethanol concentration treatment is not associated with locomotor activity. Linear regression of mean number of responses is not statistically significant (p = 0.17). (TIF) [file pone.0100894.s003.tif]

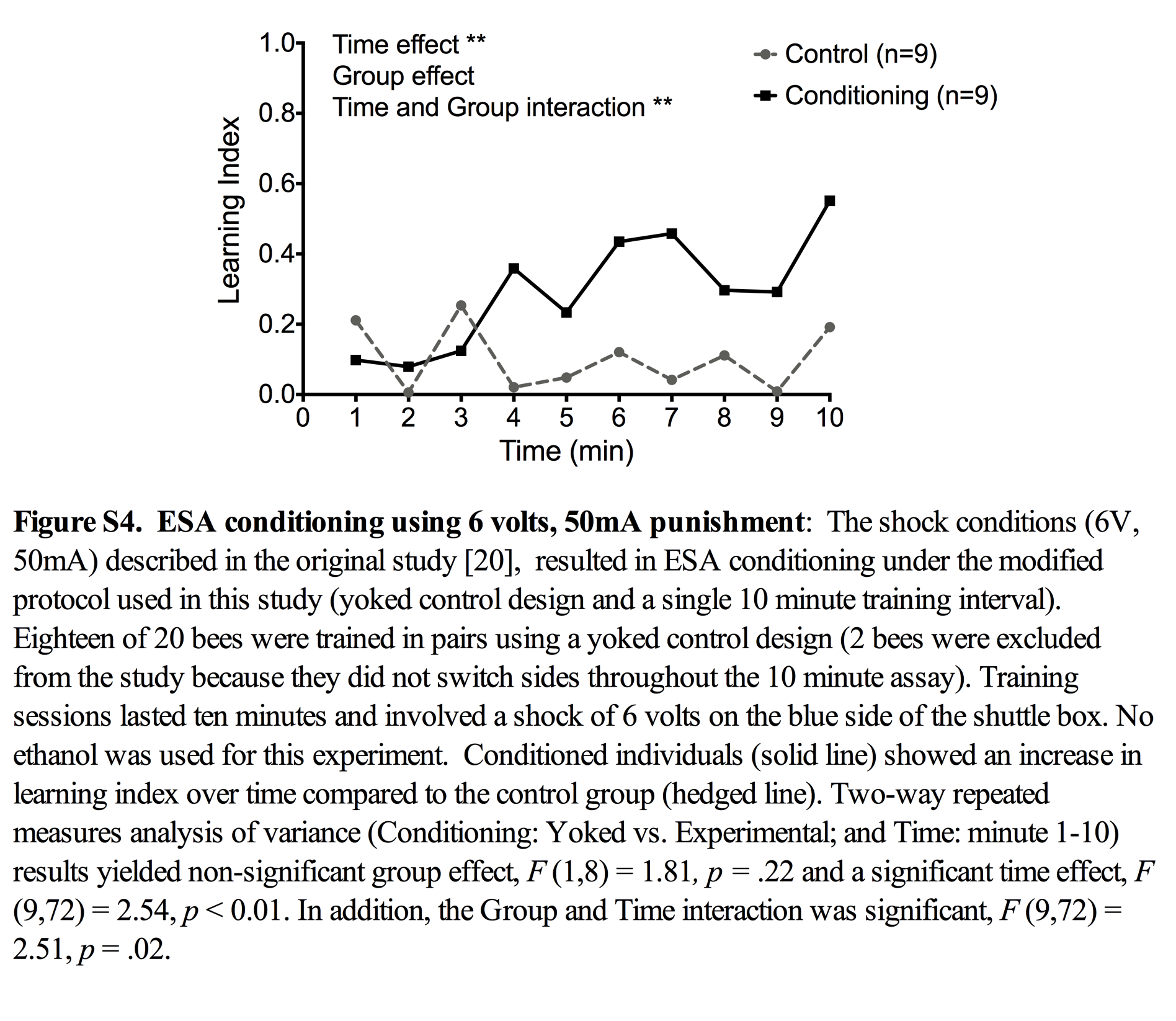

Supplement: Figure S4 — ESA conditioning using 6 volts, 50 mA punishment. The shock conditions (6 V, 50 mA) described in the original study [20], resulted in ESA conditioning under the modified protocol used in this study (yoked control design and a single 10 minute training interval). Eighteen of 20 bees were trained in pairs using a yoked control design (2 bees were excluded from the study because they did not switch sides throughout the 10 minute assay). Training sessions lasted ten minutes and involved a shock of 6 volts on the blue side of the shuttle box. No ethanol was used for this experiment. Conditioned individuals (solid line) showed an increase in learning index over time compared to the control group (hedged line). Two-way repeated measures analysis of variance (Conditioning: Yoked vs. Experimental; and Time: minute 1–10) results yielded non-significant group effect, F (1,8) = 1.81, p = .22 and a significant time effect, F (9,72) = 2.54, p<0.01. In addition, the Group and Time interaction was significant, F (9,72) = 2.51, p = .02. (TIFF) [file pone.0100894.s004.tif]

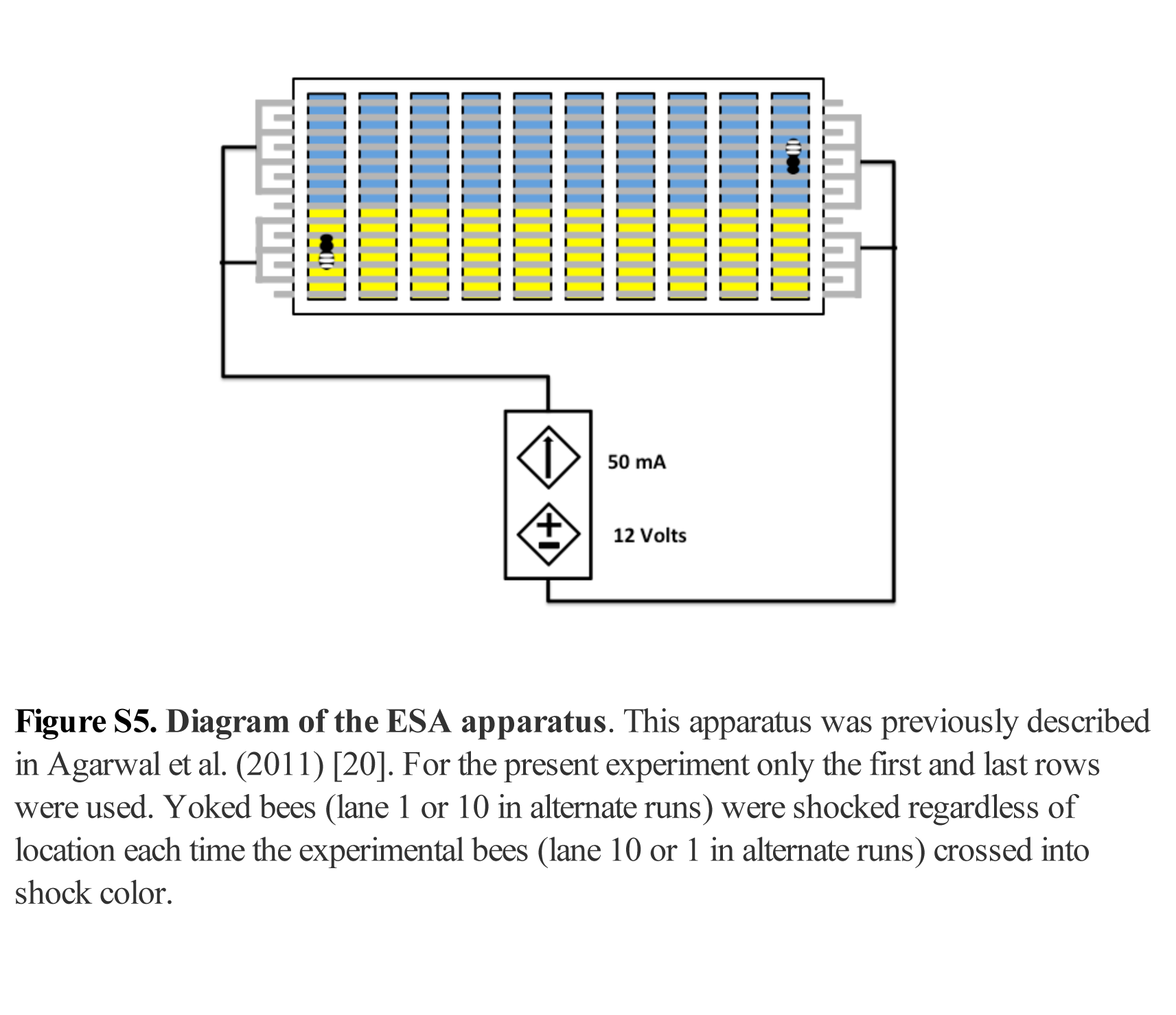

Supplement: Figure S5 — Diagram of the ESA apparatus. This apparatus was previously described in Agarwal et al. (2011) [20]. For the present experiment only the first and last rows were used. Yoked bees (lane 1 or 10 in alternate runs) were shocked regardless of location each time the experimental bees (lane 10 or 1 in alternate runs) crossed into shock color. (TIF) [file pone.0100894.s005.tif]
